# Use of feedback to improve mental number line representations in primary care clinics

**DOI:** 10.1186/s12911-018-0618-6

**Published:** 2018-06-20

**Authors:** Rachel F. Eyler, Sara Cordes, Benjamin R. Szymanski, Liana Fraenkel

**Affiliations:** 1 Department of Pharmacy Practice, University of Connecticut School of Pharmacy, 69 North Eagleville Road, Storrs, CT 06269 USA; 2 Department of Psychology, Boston College, 300 McGuinn Hall, 140 Commonwealth Ave, Chestnut Hill, MA 02467 USA; 3 Department of Chronic Disease Epidemiology, Yale School of Public Health, 60 College Street, New Haven, CT 06510 USA; 4 Department of Medicine, Section of Rheumatology, Yale University School of Medicine, 300 Cedar Street, TAC #525, New Haven, CT 06520 USA; 5 VA Connecticut Healthcare System, West Haven, CT 06516 USA

**Keywords:** Numeracy, Mental number line, Calibration

## Abstract

**Background:**

As patients become more engaged in decisions regarding their medical care, they must weigh the potential benefits and harms of different treatments. Patients who are low in numeracy may be at a disadvantage when making these decisions, as low numeracy is correlated with less precise representations of numerical magnitude. The current study looks at the feasibility of improving number representations. The aim of this study was to evaluate whether providing a small amount of feedback to adult subjects could improve performance on a number line placement task and to determine characteristics of those individuals who respond best to this feedback.

**Methods:**

Subjects from two outpatient clinic waiting rooms participated in a three phase number line task. Participants were asked to place numbers on a computerized number line ranging from 0 to 1000 in pre-test, feedback, and post-test phases. Generalized estimating equations were used to model log-transformed scores and to test whether 1) performance improved after feedback, and 2) the degree of improvement was associated with age, education level or subjective numeracy.

**Results:**

There was an overall improvement in task performance following the feedback. The average percent absolute error was 7.32% (SD: 6.00) for the pre-test and 5.63% (SD: 3.71) for the post-test. There was a significant interaction between college education and post-test improvement. Only subjects without some college education improved with feedback.

**Conclusions:**

Adults who do not have higher levels of education improve significantly on a number line task when given feedback.

## Background

Patients are increasingly asked to take an active role in decision-making at all levels of their medical care. Examples of such decisions include choosing between competing health insurance plans, deciding whether to pursue cancer screening, or choosing between competing treatment options.

Extant data suggest that numerical aptitude influences patients’ decision making. Patients with low numeracy, which can be measured either objectively (i.e., having a patient answer math problems) or subjectively (i.e., asking the patient how comfortable they feel with numbers), are at a disadvantage when making health-related decisions, as they have greater difficulty interpreting magnitudes of risk compared to those who are more numerate [1, 2]. For example, in a survey of women aged 40–50, participants overestimated the risk that they would develop and die of breast cancer within the next 10 years, with women who were lowest in objective numeracy making the largest overestimations [1]. Subjects with lower objective numeracy also tend to overestimate risks associated with medications [3]. In one survey where subjects were asked to consider a hypothetical cholesterol medication, when risk was presented numerically, 18% of the less numerate subjects (as measured by an abbreviated objective numeracy scale [4]) overestimated the risk of taking the medication, compared to only 6% in the more numerate group [3]. Research has also demonstrated that subjects with low objective numeracy might overestimate benefits of testing and procedures, as seen in one study examining women’s perceptions of the benefits related to mammography [2].

Much research has focused on presenting side effects in ways that may be more understandable to those who are low in numeracy. Indeed, visual aids such as icon arrays and bar graphs have been shown to increase understanding in patients who are low in numeracy [5–8]. However, recent data suggest that some visual aids may not be helpful to those who are also low in graphical literacy [9]. An alternative (or complementary) method may be to improve patients’ understanding of numerical magnitudes by allowing them to calibrate their mental number line.

Over- or under-estimation of risk may be linked to distortion of a subject’s intuitive representation of a mental number line [10]. This “mental number line” is traditionally observed via a number line placement task, in which individuals are asked to indicate where a given number would fall on a line with numeric endpoints (e.g., where 150 falls on a line with endpoints of 0 and 1000) [11]. Accuracy on this task has been found to correlate with mathematical achievement in children, numeracy, and more recently has been used to explore the law of diminishing marginal utility in economic decision-making [10, 12, 13].

Studies have shown that children demonstrate notable improvement in number line tasks with a small amount of feedback. For example, second graders who received feedback on the correct placement of 1–3 items had improved accuracy in 70% of number line problems, compared to just 36% for those who did not receive feedback [14]. Given this background, we sought to establish the feasibility of improving adult subjects’ numerical representations. The aim of this study was to evaluate whether providing feedback to adult subjects improves performance on a number line placement task, and whether age, education level or subjective numeracy moderates the magnitude of improvement. Because of time constraints we used the subjective numeracy scale, which has been validated against objective numeracy measures and found to predict risk recall and consistent utility assessments [15].

Given the known influence of numeracy on this task, we hypothesized that adults with higher subjective numeracy would have smaller pre-intervention errors compared to adults with lower subjective numeracy. We also hypothesized that adults with higher subjective numeracy would already perform at or near their individual peak performances, and so performance on the number line task would reveal less improvement after the feedback trials compared to adults with lower subjective numeracy.

Although subjects who are highly educated are not necessarily high in numeracy [16], amount of education is related to an individuals’ perception of magnitude [17]. Therefore we hypothesized that subjects who were more highly educated would also demonstrate smaller pre-intervention errors. Similar to subjects who are high in numeracy, we further hypothesized that adults with higher education levels would perform at or near their individual peak performance pre-intervention and so would demonstrate less improvement after the feedback compared to less educated adults.

Older adults may experience more difficulties processing information (especially numerical information) compared to their younger counterparts [18–20]. These differences are clinically important, as older adults tend to have more health-care related decisions to make. In one study, moderate to older-aged adults’ willingness to take a medication was less influenced by numerical representations of the adverse effects than younger adults [3]. Given the potential for increased numerical processing difficulties, we hypothesized that older adults would perform worse on the pre-test task (i.e., make larger errors) and feedback would be less likely to improve performance in older, compared to younger, adults.

## Methods

This study was submitted to the Yale Institutional Review Board, and was determined to be exempt. Subjects from two outpatient primary care clinic waiting rooms (both affiliated with a large academic medical center) participated in the tasks, and provided verbal consent before beginning. The research assistant approached patients consecutively. Subjects were not offered compensation for participation, and were considered eligible if they were at least 18 years of age and spoke English. Participants completed an 8-question subjective numeracy scale [21] before the tasks began. Computerized number line tasks were developed based on several studies performed with children and adults using the number line as an assessment of numerical understanding [11]. This work revealed that younger children initially place numbers along the line with logarithmic spacing, such that smaller numbers are placed further apart than larger numbers, while older children and adults (i.e., those who are more numerate) eventually place the numbers in a linear fashion. While there is debate regarding what this logarithmic-to-linear pattern may signify [11, 22, 23], it is agreed that the greatest difference in performance between individuals who are more or less numerate is observed for placements of smaller numbers, in the lower half of the range of values. As such, studies in which participants have been given feedback on their performance on the number line have determined that children show the greatest improvements in number line performance when given feedback on their placement of values in the lower half of the line, in particular, for values around 150 when placing values on a 0–1000 line [24].

**Fig. 1:**
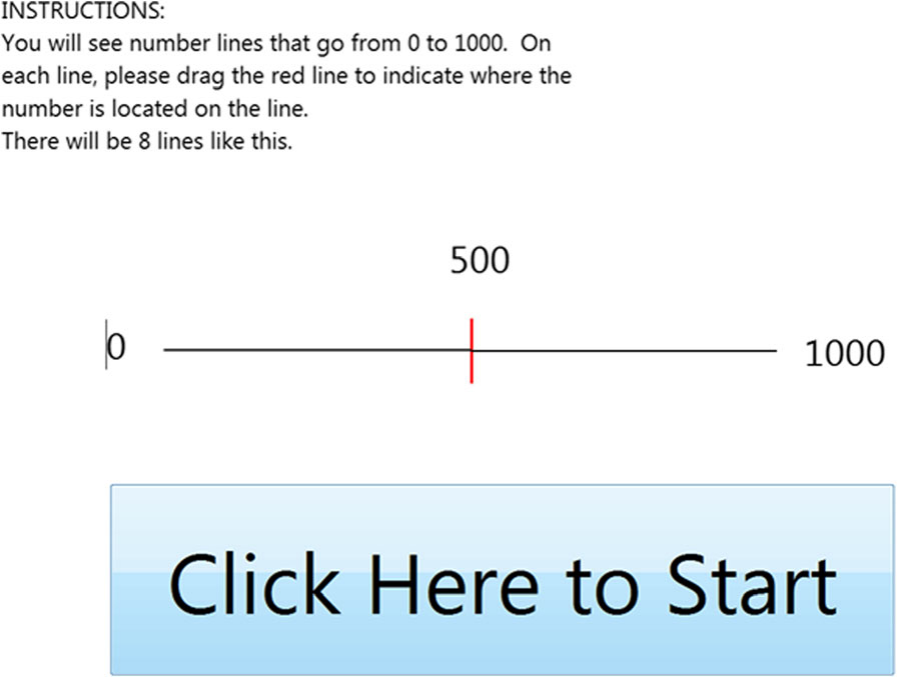
Instruction screen for the pre-test phase

**Fig. 2:**
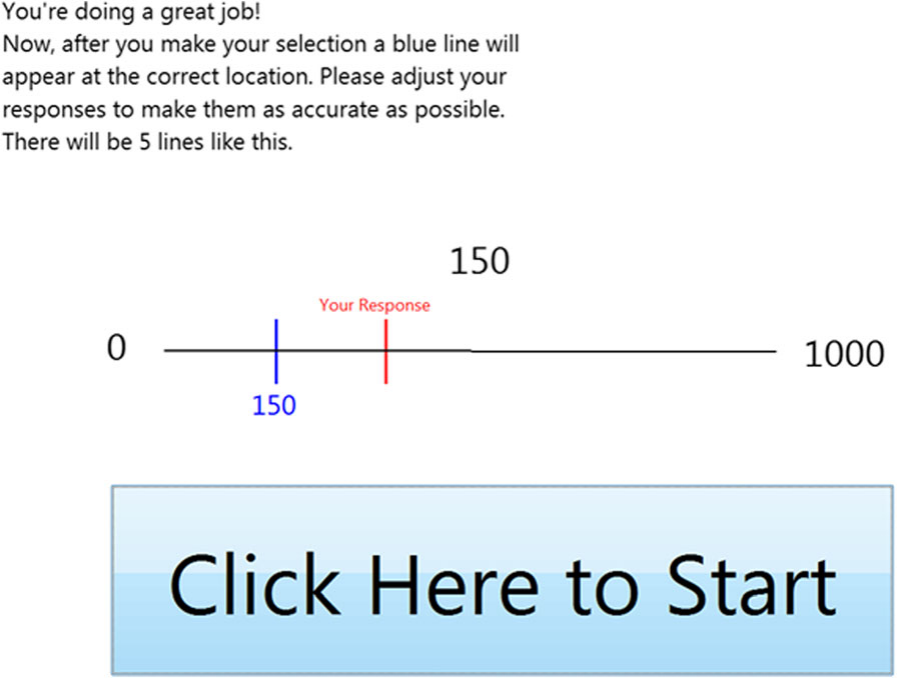
Instruction screen for feedback phase

The computerized number line tasks were used to assess each subject’s mental number line, and involved a “pre-test” phase, “feedback” phase, and “post-test” phase. In the pre-test phase, participants were asked to place eight numbers (5, 63, 119, 174, 287, 432, 641, 830, presented in random order) on a computerized number line ranging from 0 to 1000 (Fig. 1). In the feedback phase, participants were told that they were going to complete a similar task, but this time they would be able to see how “close or far” they were from the correct answer. Subjects placed an additional five numbers (169, 703, 147, 18, 156) on the number line, and the correct position was shown after each choice was made in order to provide participants with accurate feedback (Fig. 2). Finally, in the post-test phase participants were again asked to place the original eight numbers from the pre-test without feedback. The same numbers were presented to all subjects for consistency across participants, especially because there is evidence that error varies as a function of the particular values presented. At the end of the feedback phase, demographic information including age, gender, race, and level of education (less than high school, high school or equivalent, some college, college degree, or post graduate education) was collected.

Pre-test and post-test scores, reported as percent absolute error (PAE), were calculated as the average of the absolute differences between where the participant placed the number and the actual number, divided by the range of the number line (e.g., if the participant placed the number “150” on the line at the location corresponding to a value of 250, the PAE would be computed as = |250–150| / 1000 = 0.1 or 10%). Thus, lower scores represent lower error and better performance on the number line task.

Repeated measures multivariate regression was used to model PAE. The model included age, education, subjective numeracy, and whether the participant had received feedback yet. As only 9 subjects had less than a high school education, the decision was made to group individuals into those who had completed high school or less versus those who completed at least some college. Generalized estimating equations (GEE) were used to account for within-subject correlation of pretest and posttest scores. Interaction terms between feedback and age, education, and numeracy allowed the effect of feedback to vary depending on the other variables. This model allowed us to evaluate which variables were associated with overall performance (i.e., variables that had equal effect on pretest and posttest scores) and which variables were differentially associated with improvement following feedback. Log-transformation of the outcome (PAE) was performed to account for non-constant error variance. The starting model was:

$$ \log (PAE)={\beta}_0+{\beta}_1 Age+{\beta}_2 Age\times Fee dback+{\beta}_3 College+{\beta}_4 College\times Fee\mathrm{d} back+{\beta}_5 Numeracy+{\beta}_6 Numeracy\times Fee dback+{\beta}_7 College\times Numeracy+{\beta}_8 College\times Numeracy\times Fee dback+{\beta}_9 College\times Age+{\beta}_{10} College\times Age\times Fee dback+{\beta}_{11} Feedback $$

The variables in the regression model were as follows: Age was age in years, which was continuous and centered. Feedback was coded as 0 for pre-tests and 1 for post-tests. College Education was coded as 0 for people who did not complete high school and for people who only completed high school. It was coded as 1 for people who attended some college, graduated college, or had a postgraduate degree. Numeracy was the score on the subjective numeracy scale, which was continuous and centered. The dependent variable, lnPAE, was the natural log of the percent absolute error for the number line task. Using backward elimination, non-significant (*p* > 0.10) terms were removed from the model. The final model was:

$$ \log (PAE)={\beta}_0+{\beta}_3 College+{\beta}_4 College\times Feedback+{\beta}_5 Numeracy+{\beta}_{11} Feedback $$

All analyses were performed in SAS version 9.4 (SAS Institute, Inc., Cary, NC).

## Results

**Fig. 3:**
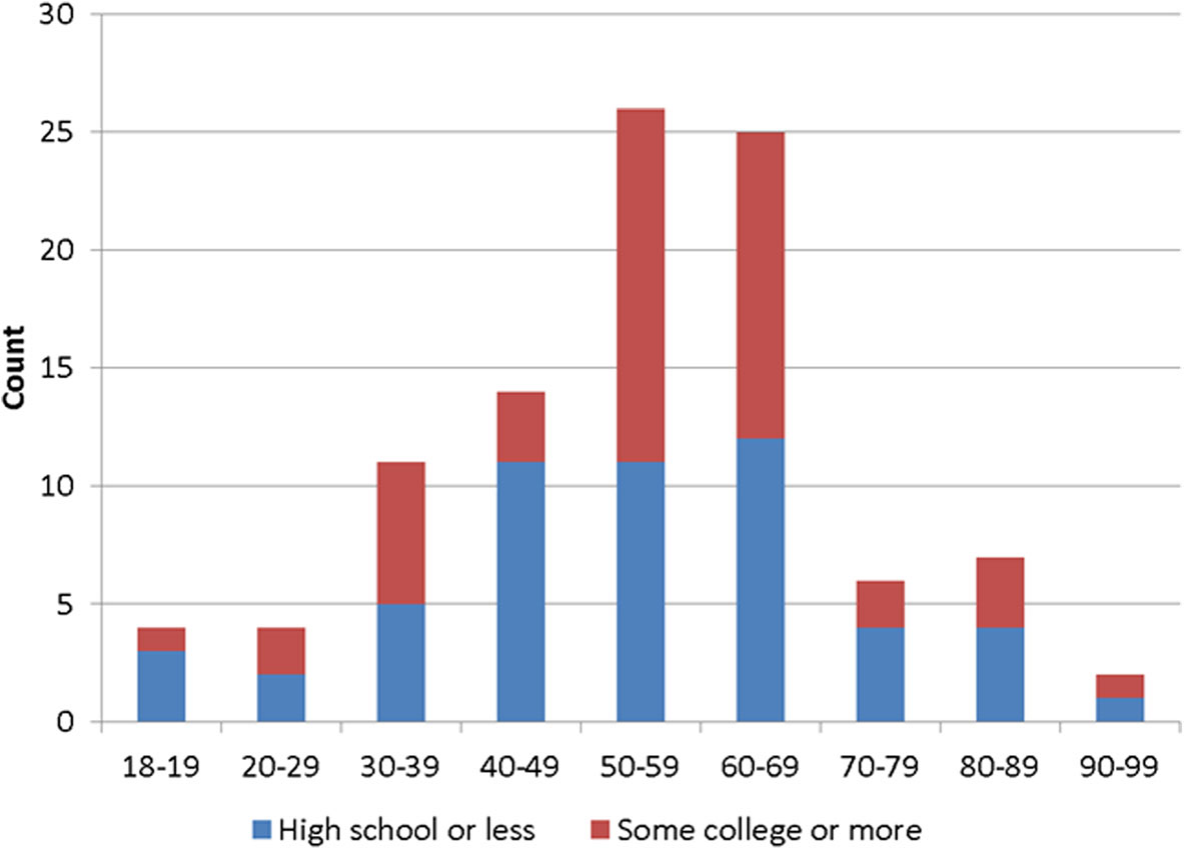
Distribution of age and education

One hundred and one subjects participated. Of these, 59 (58.4%) were female, and 46 (45.5%) had at least some college education. The mean (SD) age was 55.1 (16.8) years and the mean (SD) subjective numeracy was 4.1 (1.1) out of a possible 6. We did not find a significant association between age and education (Fisher’s exact *p* = 0.552) (Fig. 3). Thirty-seven (36.6%) participants were White, 56 (55.5%) were Black, and 8 (7.9%) were Hispanic.

There was an overall improvement (decrease in PAE) in number line task performance following the feedback training. The average PAE was 7.32% (SD: 6.00%) for the pre-test and 5.63% (SD: 3.71%) for the post-test (paired *t* = 3.90, DF = 100, *p* < 0.001).

**Fig. 4:**
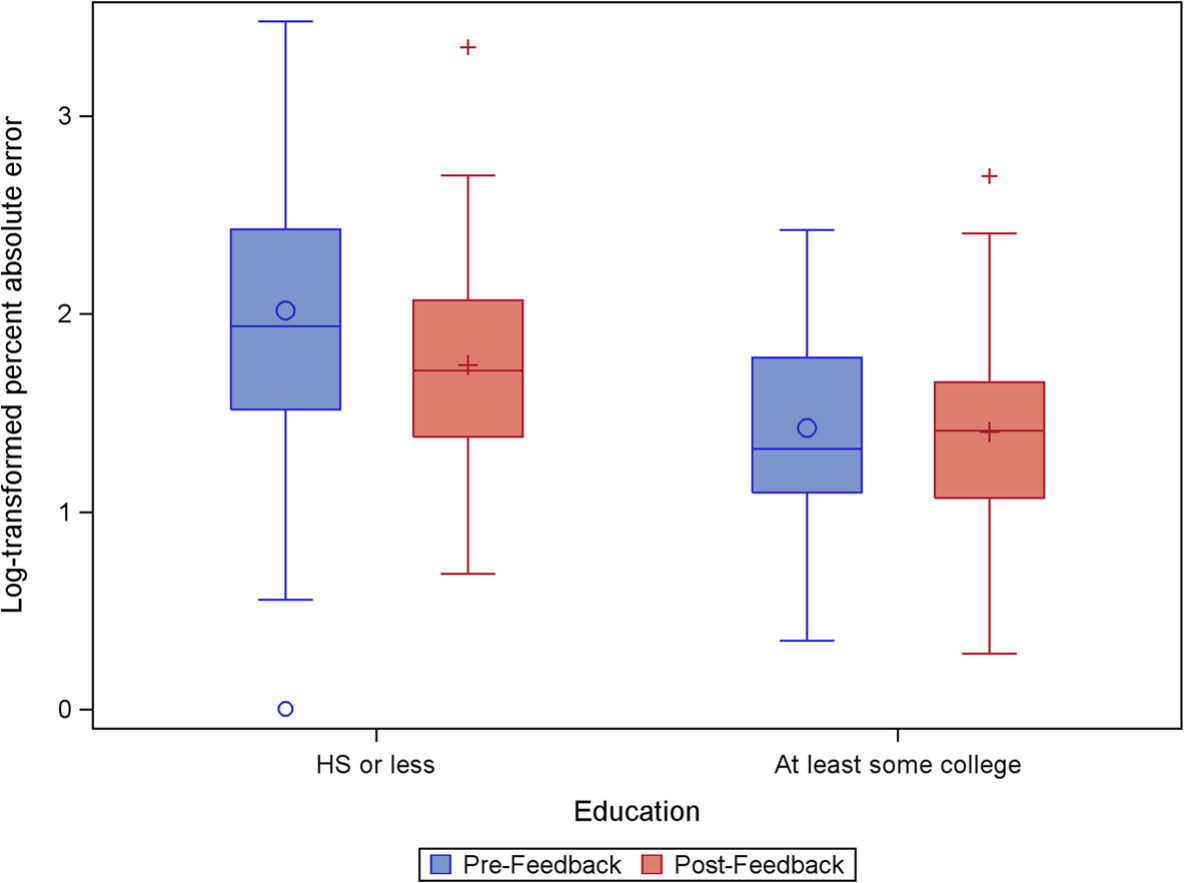
Pre- and post-test Log-transformed percent absolute error by education status

In the multivariate log-transformed GEE model (see Table 1), some college education, and higher subjective numeracy were associated with lower PAEs on both pre- and post-test tasks. Age was not associated with number line performance, and was dropped from the final model. There was an overall decrease in PAE following feedback (β = − 0.27, 95%CI: -0.41 to − 0.14). We found no interaction between subjective numeracy and feedback, suggesting that the intervention had the same effect on individuals with high and low subjective numeracy. There was, however, a significant interaction between college education and feedback (β = 0.25, 95%CI: 0.06 to 0.44). Participants with college education had lower pre-test PAEs and did not significantly improve after feedback, while those without a college education tended to have higher pre-test PAEs and improved significantly following the feedback phase (see Fig. 4).

**Table 1:** Full and reduced multivariate models of variables associated with PAE

	Full Model			Reduced Model		
Parameter	Estimate	95% Confidence Interval	*p*-value	Estimate	95% Confidence Interval	*p*-value
Intercept	1.99	(1.73, 2.25)	< 0.001	1.94	(1.73, 2.16)	< 0.001
Age	0.01	(−0.01, 0.02)	0.344			
Age x Feedback	−0.01	(−0.02, 0.00)	0.102			
College Education	−0.47	(−0.77, − 0.16)	0.002	−0.44	(− 0.72, − 0.16)	0.002
College Education x Feedback	0.31	(0.09, 0.53)	0.006	0.25	(0.06, 0.44)	0.009
Numeracy	−0.01	(−0.04, 0.02)	0.560	−0.02	(− 0.03, − 0.01)	0.004
Numeracy x Feedback	−0.00	(− 0.02, 0.02)	0.715			
College Education x Numeracy	−0.02	(−0.05, 0.02)	0.386			
College Education x Numeracy x Feedback	−0.01	(−0.03, 0.02)	0.679			
College Education x Age	−0.01	(−0.02, 0.01)	0.299			
College education x Age x Feedback	0.01	(−0.00, 0.02)	0.115			
Feedback	−0.30	(−0.46, − 0.14)	< 0.001	−0.27	(− 0.41, − 0.14)	< 0.001

Note: continuous variables were centered

In the reduced model (Table 1), the intercept of 1.94 represents the expected value of lnPAE when all other variables are 0: that is, for a non-college educated (college = 0), averagely numerate (centered numeracy = 0) person taking the pre-test (feedback = 0). The signs of the parameter estimates for college education, numeracy, and feedback are all negative, which indicates that these are all associated with smaller lnPAE. The sign for the interaction between college education and feedback is positive, which means that the benefit of feedback is diminished in people with a college education.

Considered another way, the predicted pre-test lnPAE for a person with average subjective numeracy without a college education is 1.94. The predicted post-test lnPAE for a person with average subjective numeracy without a college education is 1.94–0.27 (the main effect of feedback) = 1.67. The predicted pre-test lnPAE for a person with average subjective numeracy with a college education is 1.94–0.44 (the main effect of college) = 1.50. Finally, the predicted post-test lnPAE for a person with average subjective numeracy with a college education is 1.94–0.27 (the main effect of feedback) – 0.44 (the main effect of college) + 0.25 (the interaction between feedback and college) = 1.48.

**Fig. 5:**
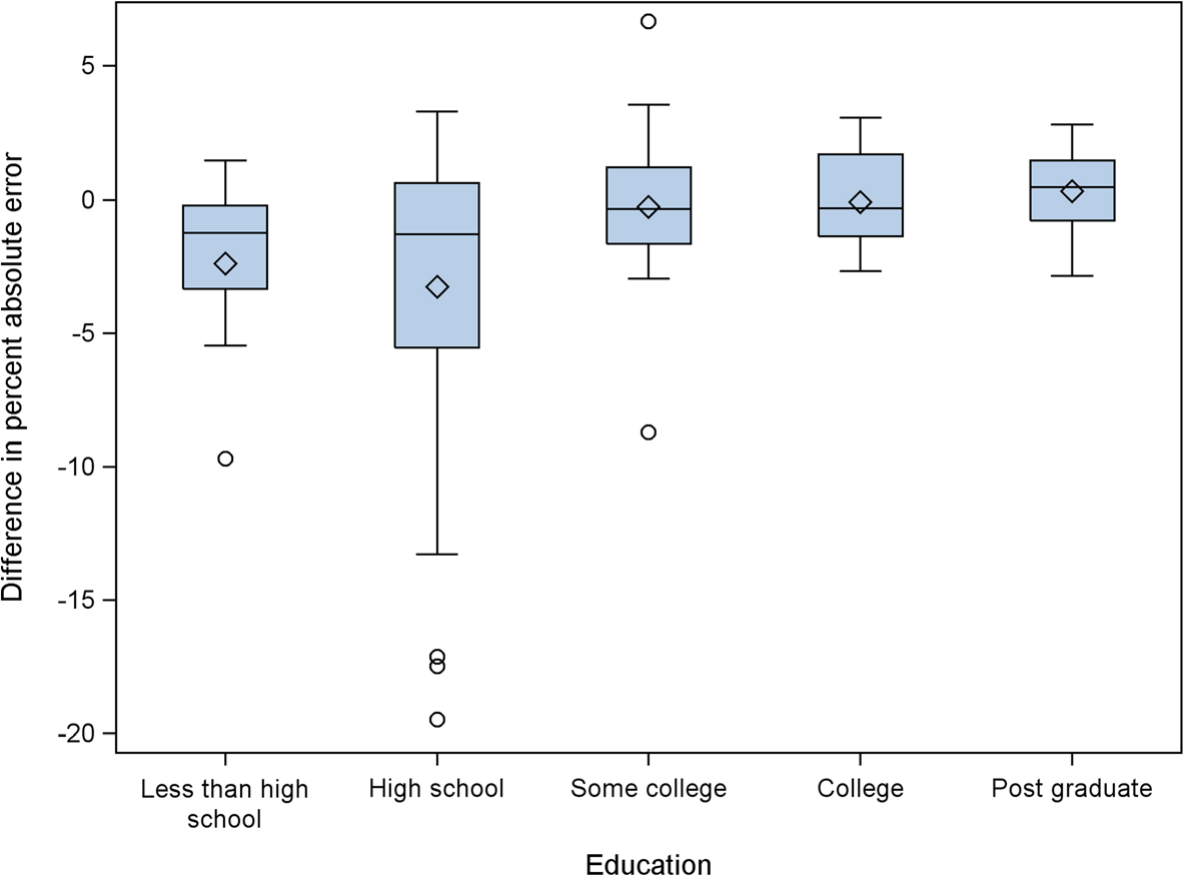
Differences in percent absolute errors (posttest – pretest) by education. *Note: Negative differences in percent absolute error indicate improved performance on the post-test

In order to see whether the observed education effect was driven by individuals at the extremes (i.e., those who had not finished high school or those with post-graduate degrees), we plotted difference in PAEs (posttest – pretest) for each of the five education groups (Fig. 5). This did not appear to be the case, as those with a high school education appear to have improved slightly more than those who had not finished high school. All three groups with at least some college education had similar differences in PAE, which were all near zero. In an unadjusted paired t-test, subjects with a high school education or less (*n* = 53) lowered their PAE by 3.12 percentage points following feedback, from 9.67 to 6.55% (*t* = 4.34, df = 52, *p* < 0.001). Subjects who had completed some college or more (*n* = 46) had no significant improvement following feedback from 4.75 to 4.68%, a difference of − 0.07 percentage points (*t* = 0.21, df = 45, *p* = 0.832).

Finally, we assessed whether the effect of the feedback was stronger for smaller (< 300) numbers on the number line, as these were the values specifically targeted by the feedback. We calculated a PAE including only the 5 numbers that fell in the first third of the number line. For participants who did not complete high school the small-value PAE was 10.18% in the pre-test and 6.10% in the post-test for a decrease of 4.08 percentage points. For participants with at least some college education, the small-value PAE was 4.64% in the pre-test and 4.47 in the post-test for a decrease of 0.17 percentage points.

## Discussion

In order for patients to make informed health-related decisions, they must be able to weigh risks and benefits. One aspect of understanding the magnitude of these risks and benefits is to understand the numerical values associated with the risks. Previous work has determined that the number line task assesses numerical magnitude understanding [14]. In this study we found that, as has been previously demonstrated in children, brief feedback significantly improves performance on a number-line task in adults without a college education.

We found significant main effects for college education and subjective numeracy on PAE, confirming our predictions that those with high numeracy and/or education would perform better on a number line task. Although these findings are not unexpected, they confirm that findings in the pediatric [17] and undergraduate population [12] can be expanded to a heterogeneous sample of adult patients in a primary care clinic who are the most likely to be making medical-related decisions. However, contrary to our prediction that older age would lead to lower number line performance, age had no effect on PAE. Younger and older participants performed equally well on the pre-test and had similar amounts of improvement following the feedback session. This may mean that cognitive inefficiencies that occur with aging have little impact on individual’s understanding of numerical magnitudes and suggests that younger and older patients alike may benefit from a more detailed demonstration of numeric magnitude.

Interestingly, the only predictor of improvement was whether or not subjects had received at least some college education, presumably because subjects with a lower education level were able to improve with the feedback, while subjects with a higher education level were already performing near their individual peak performances before feedback was given. Importantly, education is a strong predictor of objective numeracy [25], which, in turn, is a strong predictor of one’s ability to interpret medical risk information [1–3]. Our results suggest that the same individuals who are at risk for misinterpreting medical information could benefit from number line feedback training. This is motivation for further research into whether improving number line performance can also improve medical decision making itself.

Although the improvement of PAE found in our study is less than the 5–10 percentage points reported in children (who have much higher PAEs to start with) [24, 26], the improvement in PAE of 3.12 percentage points for adult subjects who did not attend college is encouraging. Additionally, subjects who did not attend college improved the most around their placement of small numbers (those < 300), with subjects improving by 4.1 percentage points when only the lowest 5 numbers were included. This finding aligns well with previous research suggesting that children and those who are low in numeracy are more likely to place numbers in a logarithmic fashion across the line (overestimating values at the beginning of the line) [11], and might explain why patients who are low in numeracy tend to overestimate risks (particularly risks with low likelihoods) associated with medications [3, 27]. In future studies we plan to assess whether this intervention may allow patients to more accurately assess the risk of side effects, so this is the area of the number line where it is most important to see the greatest impact (since these values correspond to the type of risks discussed in medical decisions).

Performance on a number line task may be a quick and easy way to identify patients who are in need of a more detailed discussion of the meanings of numbers. Findings in children do suggest that performance on a number line task is associated with measurable, real-world outcomes. Performance on the number line task has been strongly related to numeracy, math achievement, and economic decision-making [10, 12, 13]. We do not currently know if the improvements in the number line task seen with feedback can be translated into better medical decisions, but this will be an interesting area of further research. As many patients overestimate the risk of rare side effects, it is possible that giving the patients feedback regarding the actual location of the number may be beneficial. Feedback on the number line task - even with just 1–3 numbers - has not only been shown to quickly improve performance on the number line task, but also increased 7–9 year olds’ ability to categorize numbers as “very small, small, medium, big, and very big.” [24].

Future studies should evaluate whether completing a number line exercise prior to being presented with a clinical scenario could improve risk comprehension and decision making. Alternatively, patients could indicate their perceived risks of a medication on the number line, and subjects could receive real-time feedback on how accurately they charted these risks. Many subjects in this study did appear engaged during the study (even thinking of it as a challenge or game), so this method of discussing risk could present a more interactive approach to increasing risk understanding.

### Limitations

Study limitations include the small study size and self-selection among the study population, as not everyone accepted our invitation to participate. The subjects’ mental, cognitive, physical and emotional conditions in a busy waiting room could have affected their performance on this number line task, although it was reassuring that subjects were able to improve on the number line task despite these issues, as decision-making is also often made in these similar conditions.

## Conclusions

Similar to findings in the pediatric population, adults without a college education can improve on a number line task when given a brief amount of feedback. Future research should evaluate the clinical utility of a feedback intervention to aid patients in their health-related decision making.

## Abbreviations

GEE: Generalized Estimating Equations
PAE: Percent Absolute Error
